# Farnesoid X receptor is inhibited after ileum transposition in diabetic rats: its hypoglycemic effect

**DOI:** 10.7150/ijms.80563

**Published:** 2023-04-02

**Authors:** Weijie Chen, Shengnan Zhou, Jianchun Xiao, Wei Liu, Qiang Qu, Xiaodong He

**Affiliations:** Department of General Surgery, Peking Union Medical College Hospital, Chinese Academy of Medical Sciences, Shuaifuyuan 1#, Beijing 100730, P. R. China

**Keywords:** diabetes mellitus, bile acids and salts, farnesoid X receptor, ileum, glucose

## Abstract

**Background:** Aim to investigate bile acid profile changes and the Farnesoid X receptor (FXR) status after ileotransposition (IT), and reveal its possible hypoglycemic mechanism.

**Methods:** Twenty male diabetic rats were randomly assigned into the IT group and the sham IT (SH) group. Bile acid profiles were measured using an ultra-performance liquid chromatography-tandem mass spectrometry. Glucose metabolism was monitored after oral administration of FXR inhibitor and agonist. And the expression of key FXR target genes were measured.

**Results:** The levels of β-muricholic acid (*P =* 0.047), tauro-α-muricholic acid and tauro-β-muricholic acid (*P* < 0.001) in plasma in the IT group were higher than those in the SH group, and the levels of taurocholic acid (*P =* 0.049) and turoursodeoxycholic acid (*P =* 0.030) were lower than those in the SH group. After inhibition of intestinal FXR, the glucose metabolism in the SH group was improved. When FXR agonist was given, the blood glucose level was increased in both groups. After sacrifice, the levels of glycoursodeoxycholic acid, tauro-α-muricholic acid and tauro-β-muricholic acid in liver and ileum tissues were higher than those in the SH group (*P* < 0.05), the level of α- muricholic acid (*P <* 0.001) in liver tissues were lower than that in the SH group. Moreover, the expression of CYP7A1 mRNA (*P* < 0.001) and FGF15 mRNA (*P* = 0.001) in the IT group was significantly higher, and the expression of PEPCK mRNA (*P* = 0.004), SREPB1c mRNA (*P* = 0.005) and SRB1 mRNA (*P* = 0.001) were significantly lower than that in the SH group.

**Conclusions:** We demonstrate a remarkable heterogeneity of BA profiles after IT, FXR activation might has a detrimental effect on glucose metabolism.

## Introduction

The prevalence of Type 2 diabetes mellitus (T2DM) is steadily rising worldwide, that brings many health problems leading to high medical care costs, poor quality of life and increased mortality 1. Ileum transposition (IT) is a traditional metabolic surgery, that can lead to durable improvement of T2DM 2. It is proposed that early exposure of food to the interposed ileum stimulates signal molecules including many peptides, hormones and bile acids, which could increase insulin secretion and improve insulin resistance and glucose metabolism 3, 4. However, the defined mechanism is too complex, because the metabolic surgery seems to be a “one-size-fits-all” solution. The real mechanism orchestrates functional fine tuning of many identified and unidentified factors, and the molecular mechanisms need more investigations. The clearer the mechanism of diabetic improvement after metabolic surgery investigates, the better the alternative less invasive treatment strategy will be explored.

The IT procedure is an ideal surgical method to explore the mechanism of glucose improvement, as it merely involves intestinal reconstruction and has no digestive juice bypass. The surgery transposes a part of distal ileum into the proximal jejunum without other gastrointestinal reconstructions and other organs directly involved. Many observations reported an increased level of bile acids (BAs) after IT 5. The roles of BAs and their receptors are also widely recognized in recent years. They are not only involved in lipid decomposition and absorption, but also, they could act as signaling molecules to improve glucose metabolism 6. They can bind to farnesoid X receptor (FXR), a kind of cellular receptors, to increase insulin secretion, inhibit gluconeogenesis and enhance insulin sensitivity 7, 8. In addition, the ileum actively reabsorbs approximately 85% of BAs. Surgery would disrupt BA reabsorption in the ileum and gut microbial communities which lead to BA changes 9. We verified that IT surgery procedures could accelerate the enterohepatic circulation rate of BAs and elevate plasma total BA levels 10. However, the detailed BA profiles after IT is undefined.

FXR, highly expressed in the ileum, seems to exert different effects on glucose metabolism 11. Fexaramine and Obeticholic acid are kinds of FXR agonists which were found to improve insulin resistance in obese mice 12, 13. While oral administration of FXR agonist GW4064 was found to exacerbate weight gain, glucose intolerance and dyslipidemia in obese and insulin-resistant mice 14. There were few reports about the role or the status about FXR in the glucose improvement after IT. Aim to investigate changes of BA profiles and roles of FXR after IT, we performed surgery on nonobese rats. The possible effect of BA and its' receptors on glucose metabolism might provide a potential treatment for T2DM, the less invasive target medicines other than surgical procedures might be exploited.

## Animals and Methods

### Animals

In our study, twenty 12-week male Goto-Kakizaki (GK) rats (Shanghai, P. R. China) were purchased to perform rat models. GK rats is a kind of T2DM model, could spontaneously develop insulin resistance and nonobese diabetes because of the accumulation of multiple variations 15. This can reduce impacts of body weight loss by IT, and it is relatively easier and widely used to investigate the potential mechanism of diabetic improvement. Each GK rats was housed separately in metabolic cages in a constant environment (22 °C temperature and 50% humidity) with a 12-hour light-dark cycle. It was fed a rat chow diet (5% fat by kcal, 65% carbohydrate by kcal, 30% protein by kcal, Fubo Biotechnology Co. LTD, P. R. China) before surgery. After 1- week acclimation, twenty rats were randomly divided into two groups: the IT group and the sham IT (SH) group. The sample size was calculated by previous results of bile acid changes. All experiments protocols were approved by the Ethics Committee of our hospital (XHDW-2020-029).

### Surgical procedures

**Figure 1:**
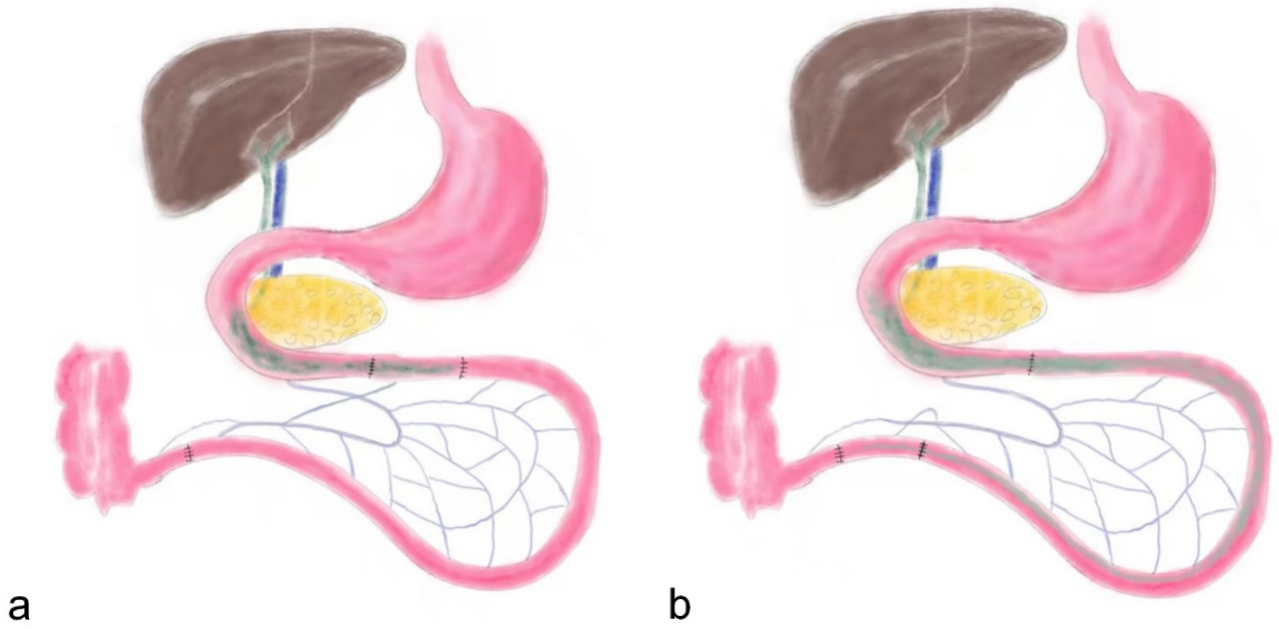
the schematic diagram of ileum transposition and sham surgery. a, the schematic map of ileum transposition. The distal ileum was transposed into the proximate intestine, and the bile acids are mainly reabsorbed in ileum, so the enterohepatic circulation of bile acids could be accelerated. b, the sham surgery involved same incisions and transections at the jejunum and ileum to simulate the trauma of ileum transposition. IT, ileum transposition. SH, sham ileum transposition.

Before surgery both groups of rats were given a nonresidue diet (15% fat by kcal, 56% carbohydrate by kcal, 26% protein by kcal, Abbott, USA) for 2 days. Subsequently, the rats were fasted overnight (12 hours), and anesthetized by peritoneal injection of Pentobarbital Sodium (30-40 mg Pentobarbital Sodium per 1 kg body weight) and Ketamine (30-40 mg Ketamine per 1 kg body weight). As previously described, the IT surgical procedure dissects an approximate 10 cm distal ileum and transposes it into the jejunum approximate 10 cm to the Treitz ligament (Figure 1) 2. Abdominal wall was closed using simple, interrupted 3-0 polypropylene sutures. The SH group rats received same incisions and transections at the jejunum and ileum to simulate the trauma of IT procedure, but no ileum transposition was performed. The jejunum and ileum were anastomosed *in situ*.

### Food intake and bodyweight

All rats were fasted and deprived of water within 12 hours after surgery. The rats started to drink 12 hours after surgery and were fed with a nonresidue diet for 10 days. Subsequently, the rat chow diet was maintained up to the endpoint measurements. Two group rats were pair-fed to maintain the same amount of food intake between the two groups. This was aimed to diminish the potential impact of food on glucose metabolism. In order to assess the appetite and real need of each rats, the maximum food intake in 24 hours was weighted. The maximum food intake was the intaking amount without restriction from 8 am to 8 am the next day. The body weight was measured at 8 o'clock after an overnight fast using an electronic scale.

### Glucose metabolism

Fasting plasma glucose (FPG) levels were tested using a glucometer (Roche, Mannheim, Germany) at 8 o'clock after an overnight fast. Blood was taken from the caudal vein. Moreover, oral glucose tolerance tests (OGTTs) were performed before surgery and at postoperative weeks 4, 12, and 20 16. After fasted overnight, all rats were fed a bolus of glucose solution (2 g glucose per 1 kg body weight). Blood glucose was tested at 15, 30, 60, and 120 minutes. The area under the curve (AUC) of the OGTT (AUC_OGTT_) was calculated by trapezoidal integration.

### Bile acid profiles

To identify changes of BA profiles after IT, 17 kinds of BAs, such as α-muricholic acid (α-MCA), β-muricholic acid (β-MCA), cholic acid (CA), chenodeoxycholic acid (CDCA), deoxycholic acid (DCA), hyodeoxycholic acid (HDCA), glycocholic acid (GCA), glycochenodeoxycholic acid (GCDCA), glycodeoxycholic acid (GDCA), glycoursodeoxycholic acid (GUDCA), lithocholic acid (LCA), tauro-α-muricholic acid (T-α-MCA), tauro-β-muricholic acid (T-β-MCA), taurocholic acid (TCA), turoursodeoxycholic acid (TDCA), taurohyodeoxycholic acid (THDCA) and tauroursodeoxycholic acid (TUDCA) were measured in samples.

All rats were fasted overnight and given 20% Intralipid fluid solution (Fresenius Kabi SSPC, Wuxi, P. R. China) orally at postoperative week 16. At 1 hour after feeding, blood was taken from the caudal veins. Quantitative detection of targeted BAs was performed on an ultra-performance liquid chromatography-tandem mass spectrometry (UPLC-MS) platform (Waters, Milford, MA, USA). The procedure was described in previous reports 17.

### Inhibition of FXR

Glycine-β-muricholic acid (Gly-β-MCA) is an oral high-affinity FXR inhibitor, that could selective block intestinal FXR 18. At postoperative week 18, each rat was given 10 mg Gly-β-MCA (Med Chem Express, Shanghai, P. R. China) per kilogram of body weight every day for 1 week, and fasting glucose levels and OGTT results were measured as described above.

### Activation of FXR

GW4064 is an oral FXR agonist 19, was bought from Med Chem Express (Shanghai, P. R. China). At postoperative week 19, each rat was given 20 mg GW4064 per kilogram of body weight every day for 1 week. Then the fasting glucose and OGTT were measured.

### Samples collection

At the end of postoperative week 20, all rats were sacrificed after anesthesia. Liver and ileum were collected within 30 minutes. About 100 mg fresh liver or 100 mg ileum samples were homogenized using 1 ml physiological saline. Ten microliters of internal standard and 1 ml cold alkaline acetonitrile were added. The mixture was centrifuged at 14800 rounds per minute and 4 ℃ for 10 minutes, reconstituted in 100 μl of methanol and deionized water (v:v, 85:15), and centrifuged at 14800 rounds per minute and 4 ℃ for 10 minutes 17. The supernatant was collected to investigate bile acid profiles by UPLS-MS.

### Quantitative real-time polymerase chain reaction (qPCR)

The expression of key FXR target genes in liver and ileum were measured by qPCR. Total RNA was extracted from hepatic tissues and ileal tissues using MiniBEST Universal RNA Extraction Kit (TaKaRa Bio. Inc., Dalian, P. R. China), and reverse transcribed to first-strand cDNA using an iScript™ cDNA synthesis kit (Bio-Rad, CA, USA). Then, an UltraSYBR Mixture kit (Thermo Scientific, USA) was used for qPCR analysis on an Applied Biosystems 7500 Fast Dx Real-Time PCR System (Applied Biosystems, USA) using CYP7A1 primer (forward: 5ʹ-CTCTAAATGCCCTGCAGATGA-3ʹ, reverse: 5ʹ-GGCACGGCTAATGATTCTCT-3ʹ), FGF15 primer (forward: 5ʹ-AAGTGGAGTGGGCGTATTGT-3ʹ, reverse: 5ʹ-AGTGGACCTTCATCCGACAC-3ʹ), PEPCK primer (forward: 5ʹ-GCCTGTGGGAAAACCAACCT-3ʹ, reverse: 5ʹ-CACCCACACATTCAACTTTCCA-3ʹ), SRB1 primer (forward: 5ʹ-ATGGTACTGCCGGGCAGAT-3ʹ, reverse: 5ʹ-CGAACACCCTTGATTCCTGGTA-3ʹ), and SREPB1c primer (forward: 5ʹ-ATGGATTGCACATTTGAAGACATGCTTCAG-3ʹ, reverse: 5ʹ-CCTGGCGATGGCTGTGGTGCTG-3ʹ) to obtain the value of Ct 20-24. We used GAPDH (forward primer: 5ʹ-GCAAGTTCAACGGCACAG-3ʹ, reverse primer: 5ʹ-GCCAGTAGACTCCACGACAT-3ʹ) as the internal reference gene. The relative expressions of interesting genes were quantified by the method of 2^-ΔΔCt^.

### Statistical analysis

Quantitative data are expressed as the mean ± standard deviation. The data of both groups was taken a homogeneity test for variance in multiplicate samples by the means of Bartlett. Considering the number of measures, the data distribution and the homogeneity of variances, differences of continuous variable before and after surgery were calculated by repeated-measures analysis of variance and paired t test using SPSS version 26.0 (SPSS, Chicago, IL, USA), and differences between the IT group and SH group were calculated by Analysis of Variance and t test. The *P* value < 0.05 was considered statistically significant.

## Results

### Rat models

All surgeries and procedures were performed successfully in rat models. During the postoperative recovery period, An IT rat died at week 3, and an SH rat died at week 4. The postmortem examination was performed to clarify the diagnosis. The intestinal obstructions and abdominal adhesion were found in the two rats. No other complications were found.

### Food intake amount and bodyweight

**Figure 2:**
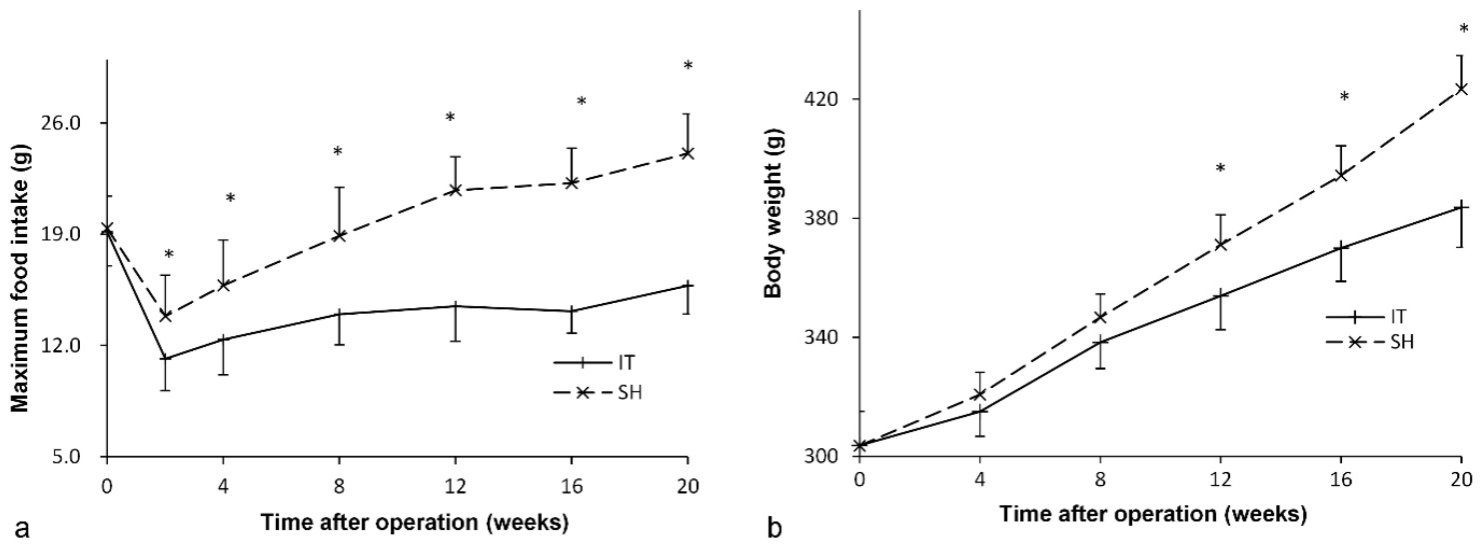
Maximum food intake curve and body weight curve of rats. a, Maximum food intake curve of two group rats. The maximum food intake in 24 hours of the IT group was less than that of the SH group after surgery (*P* < 0.001). b, Body weight curve of two group rats. From 12 weeks after surgery, the body weight of the IT group was less than that of the SH group (*P* < 0.001). IT, ileum transposition. SH, sham ileum transposition.

Before surgery, the food intake amount of two groups was similar (*P* = 0.816). After surgery, food intake in the IT group decreased immediately and recovered slowly at postoperative week 2 (Figure 2). The maximum food intake amount in the IT group was 15.8 ± 1.8 g at postoperative week 20, which was less than that before surgery (19.2 ± 2.1 g,* P* < 0.001). And the maximum food intake amount in the IT group was less than that in the SH group at postoperative week 20 (15.8 ± 1.8 g vs. 24.1 ± 2.5 g, *P* < 0.001).

Similarly, there was no significant difference in body weight between two groups before surgery (*P* = 0.976, Figure 2). After surgery, the body weight of the IT group increased from 303.7 ± 11.7 g before surgery to 383.7 ± 13.5 g at postoperative week 20 (*P* < 0.001). The body weight of the SH group also increased from 303.6 ± 11.5 g to 423.4 ± 11.3 g (*P* < 0.001), so it was more than that of the SH group at postoperative week 20 (423.4 ± 11.3 g vs. 383.7 ± 13.5 g, *P* < 0.001).

### Effect on glucose metabolism

**Figure 3:**
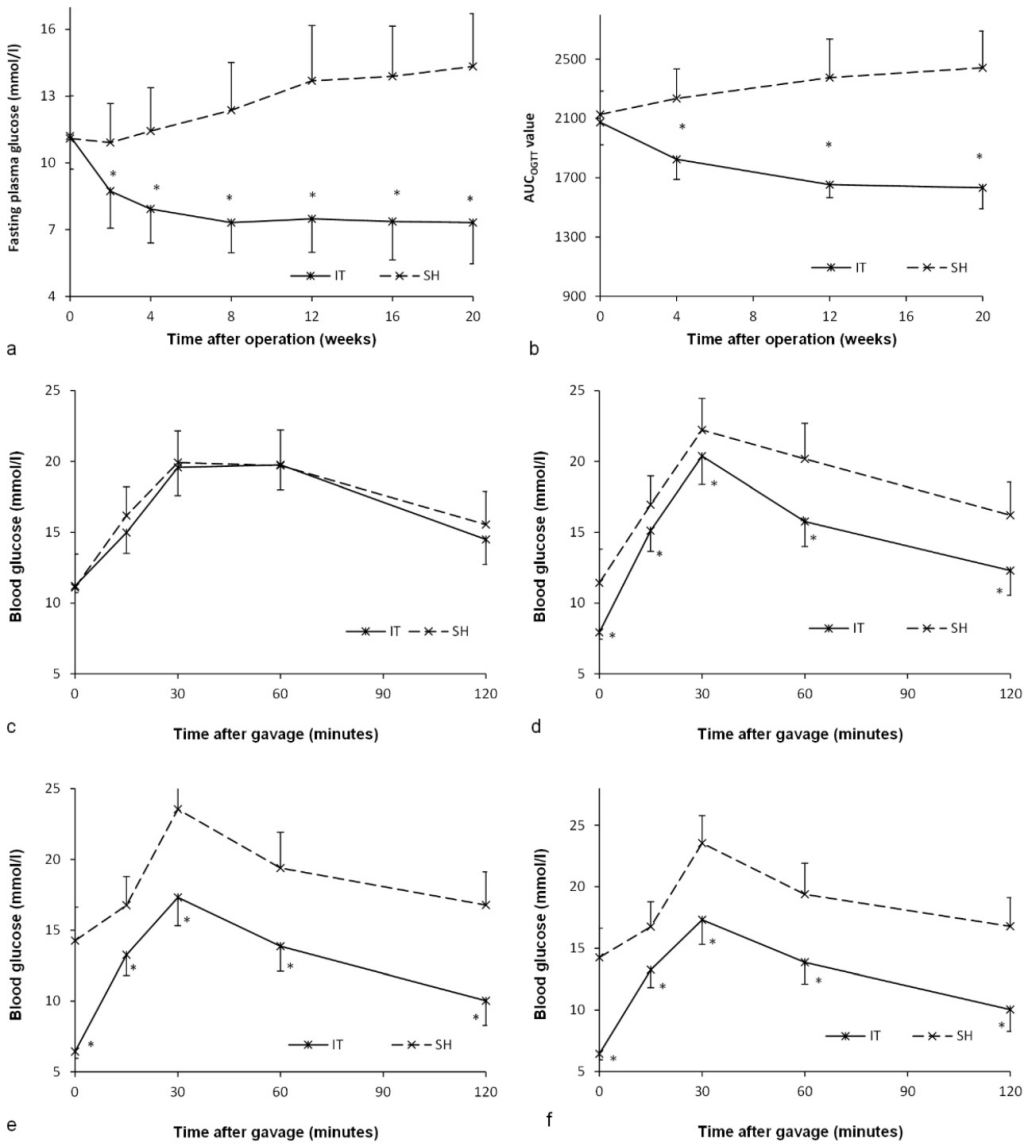
Glucose metabolism of two groups. a, Fasting glucose curve of the two groups. The lower fasting glucose level was observed in the IT group after surgery (*P* < 0.001). b, AUC_OGTT_ value during the postoperative period. The AUC_OGTT_ value of the IT group was less than that of the SH group after surgery (*P* < 0.001). c, Glucose curve during the OGTT before surgery. d, Glucose curve during the OGTT at postoperative week 4. e, Glucose curve during the OGTT at postoperative week 12. f, Glucose curve during the OGTT at postoperative week 20.

Before surgery, there was no significant difference between the IT group and the SH group in the level of FPG (*P* = 0.887) or the AUC_OGTT_ value (*P* = 0.448, Figure 3)_._ After surgery, the FPG and AUC_OGTT_ values in the IT group decreased significantly, while the FPG and AUC_OGTT_ values in the SH group increased significantly. The FPG level in the IT group decreased from 11.2 ± 1.5 mmol/l to 7.3 ± 1.9 mmol/l at postoperative week 20 (*P* < 0.001), and the AUC_OGTT_ value decreased from 2073.5 ± 150.8 mmol/l·min to 1632.1 ± 141.3 mmol/l·min (*P* < 0.001). While the FPG level in the SH group increased from 11.1 ± 1.9 mmol/l to 14.3 ± 2.4 mmol/l at postoperative week 20 (*P* = 0.001), and the AUC_OGTT_ value increased from 2126.9 ± 157.0 mmol/l·min to 2442.3 ± 246.7 mmol/l·min (*P* = 0.001). Therefore, the FPG in the IT group (7.3 ± 1.9 mmol/l) was remarkably lower than that in the SH group at postoperative week 20 (14.3 ± 2.4 mmol/l, *P* < 0.001). And the AUC_OGTT_ value in the IT group (1632.1 ± 141.3 mmol/l·min) was less than that in the SH group (2442.3 ± 246.7 mmol/l·min, *P* < 0.001).

### Bile acid profiles

**Figure 4:**
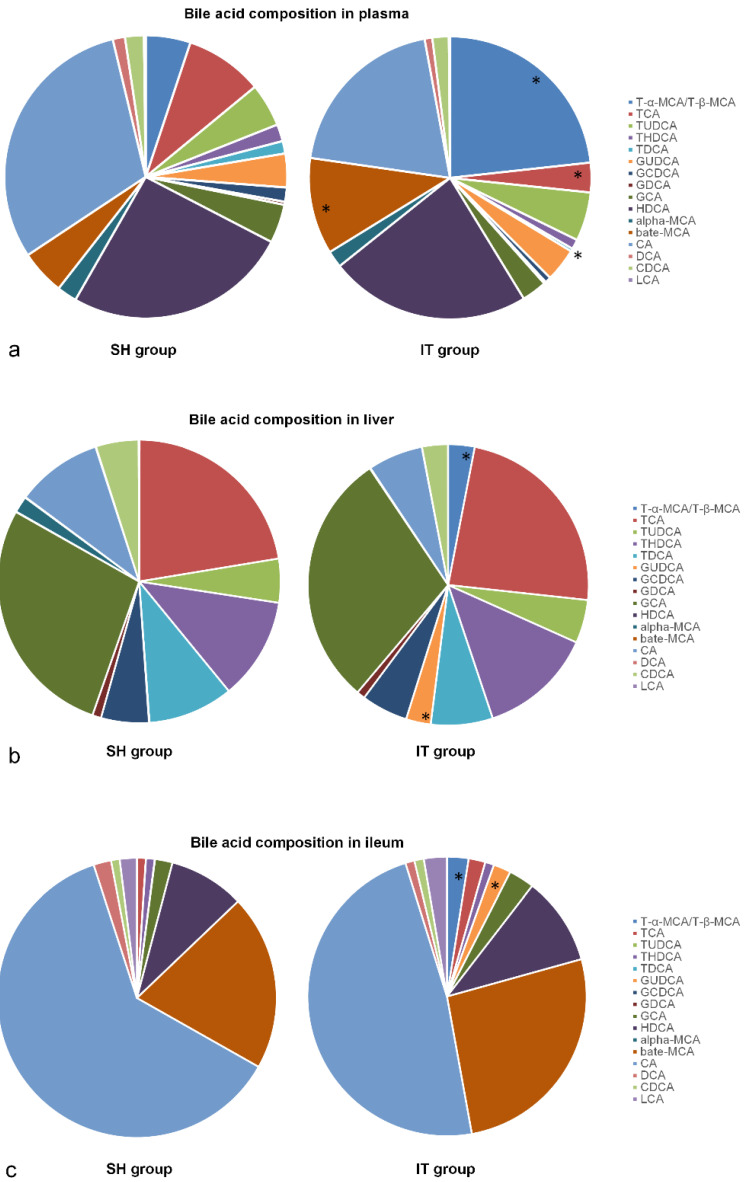
Bile acid profile changes after IT. a, Bile acid profiles in plasma after IT. The level of β-MCA, T-α-MCA and T-β-MCA in the IT group was higher than that in the SH group, and the level of TCA and TDCA was lower than that in the SH group (*P* < 0.05). b, Bile acid profiles in liver after IT. The level of GUDCA, T-α-MCA and T-β-MCA in liver tissues in the IT group was higher than that in the SH group, and the level of α-MCA was lower than that in the SH group (*P* < 0.05). c, Bile acid profiles in ileum after IT. The levels of GUDCA, T-α-MCA and T-β-MCA in the IT group were higher than those in the SH group (*P* < 0.05). α-MCA, α-muricholic acid. β-MCA, β-muricholic acid. CA, cholic acid. CDCA, chenodeoxycholic acid. DCA, deoxycholic acid. HDCA, hyodeoxycholic acid. LCA, lithocholic acid. GCA, glycocholic acid. GCDCA, glycochenodeoxycholic acid. GDCA, glycodeoxycholic acid. GUDCA, glycoursodeoxycholic acid. T-α-MCA, tauro-α-muricholic acid. T-β-MCA, tauro-β-muricholic acid. TCA, taurocholic acid. TDCA, turoursodeoxycholic acid. THDCA, taurohyodeoxycholic acid. TUDCA, tauroursodeoxycholic acid. IT, ileum transposition. SH, sham ileum transposition.

At postoperative week 16, all 17 kinds of BAs in plasma samples were analyzed. The composition of BAs in the IT group was different from that in the SH group. The amount of β-MCA (448.5 ± 389.0 ng/ml vs. 151.7 ± 142.9 ng/ml, *P =* 0.047), T-α-MCA and T-β-MCA (945.2 ± 826.7 ng/ml vs. 151.5 ± 50.8 ng/ml, *P* < 0.001) in the IT group was more than that in the SH group, and the amount of TCA (137.6 ± 77.1 ng/ml vs. 263.7 ± 159.5 ng/ml, *P =* 0.049) and TDCA (13.4 ± 5.1 ng/ml vs. 42.0 ± 35.7 ng/ml, *P =* 0.030) was less than that in the SH group (Figure 4).

After sacrifice, liver and ileum samples were collected at postoperative week 20. The BA profiles are showed in Figure 4. The amount of GUDCA (1687.7 ± 2352.3 ng/ml vs. 16.8 ± 17.2 ng/ml, *P =* 0.049), T-α-MCA and T-β-MCA (1800.0 ± 1857.5 ng/ml vs. 15.5 ± 14.0 ng/ml, *P =* 0.011) in liver tissues in the IT group was more than that in the SH group, and the amount of α-MCA (26.8 ± 29.9 ng/ml vs. 535.1 ± 314.3 ng/ml, *P <* 0.001) was less than that in the SH group. In ileal tissue samples, the amount of GUDCA (12673.2 ± 7011.8 ng/ml vs. 62.2 ± 88.9 ng/ml, *P <* 0.001), T-α-MCA and T-β-MCA (15645.0 ± 17267.4 ng/ml vs. 111.0 ± 179.7 ng/ml, *P =* 0.016) in the IT group was more than that in the SH group (Figure 4).

### Inhibition of FXR

**Figure 5:**
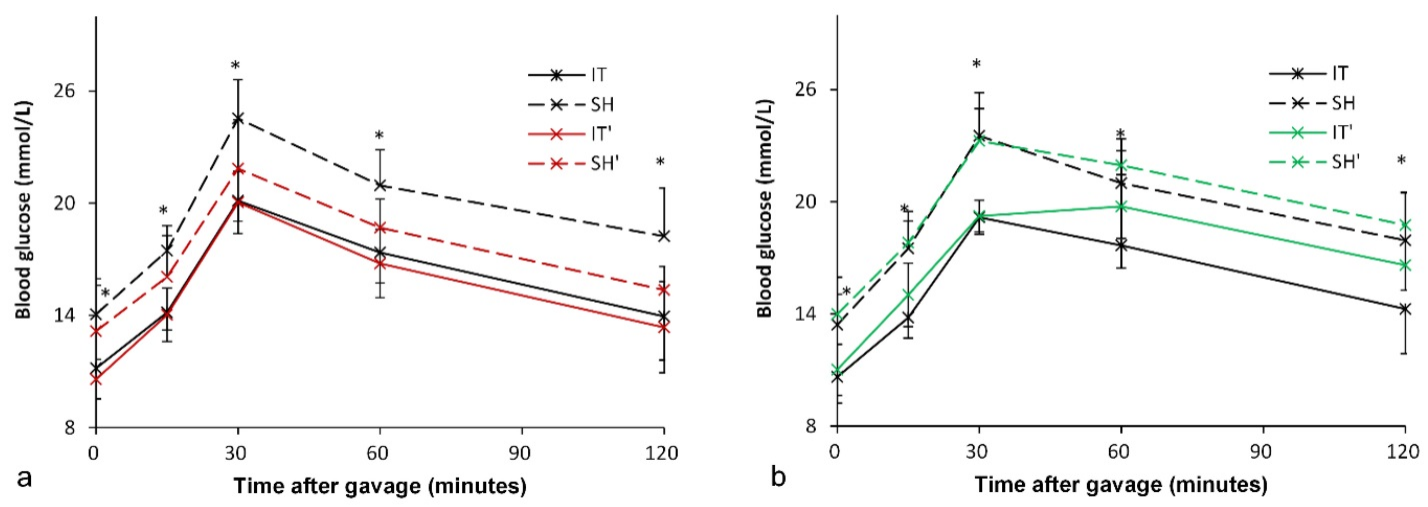
Blood glucose levels after FXR inhibition and activation. a, Glucose curve after oral FXR inhibitor administration. The glucose level and the AUC_OGTT_ value in the SH group rats decreased after FXR inhibition (*P* = 0.049). There was no significant difference in the glucose level and the AUC_OGTT_ value after FXR inhibition in the IT group (*P* = 0.081). b, Glucose curve after oral FXR agonist administration. The glucose level (*P* = 0.003) and the AUC_OGTT_ value (*P* = 0.011) in the IT group rats increased after FXR activation. The FPG level increased after FXR agonist administration (*P* = 0.013), and there was no significant difference in the AUC_OGTT_ value before and after FXR activation. FXR, farnesoid X receptor. IT, ileum transposition. SH, sham ileum transposition. IT', ileum transposition group rats after oral drug administration. SH', sham ileum transposition group rats after oral drug administration.

At postoperative week 18, all rats were given Gly-β-MCA, an intestinal FXR inhibitor, for 1 week. An improvement in glucose metabolism in the SH group was observed. In the SH group, the FPG level decreased from 14.1 ± 1.9 mmol/l to 13.1 ± 2.5 mmol/l (*P* = 0.049), and the AUC_OGTT_ value decreased from 2409.5 ± 216.8 mmol/l·min to 2133.3 ± 191.3 mmol/l·min (*P* < 0.001). In the IT group, there was no significant difference in the FPG level (11.2 ± 1.7 mmol/l vs. 10.6 ± 1.0 mmol/l, *P* = 0.081) before and after Gly-β-MCA administration, or no significant difference in the AUC_OGTT_ value (1949.2 ± 167.5 mmol/l·min vs. 1896.9 ± 188.5 mmol/l·min, *P* = 0.057) before and after Gly-β-MCA administration (Figure 5).

Although intestinal FXR was blocked, the FPG level in the IT group was still lower than that in the SH group (10.6 ± 1.0 mmol/l vs. 13.1 ± 2.5 mmol/l, *P* = 0.010), and the AUC_OGTT_ value was also lower than that in the SH group (1896.9 ± 188.5 mmol/l·min vs. 2133.3 ± 191.3 mmol/l·min, *P* = 0.018).

### Activation of FXR

At postoperative week 19, all rats were given FXR agonist for 1 week. In the IT group, the FPG level increased from 10.6 ± 1.4 mmol/l to 11.0 ± 1.4 mmol/l (*P* = 0.003), and the AUC_OGTT_ value increased from 1941.0 ± 150.1 mmol/l·min to 2127.5 ± 153.6 mmol/l·min (*P* = 0.011). In the SH group, the FPG level increased from 13.4 ± 1.6 mmol/l to 14.0 ± 2.0 mmol/l (*P* = 0.013), and there was no significant difference in the AUC_OGTT_ value (2375.3 ± 221.6 mmol/l·min vs. 2445.0 ± 148.1 mmol/l·min, *P* = 0.224) before and after FXR activation (Figure 5).

The FPG level in the IT group was still lower than that in the SH group (11.0 ± 1.4 mmol/l vs. 14.0 ± 2.0 mmol/l, *P* = 0.002), and the AUC_OGTT_ value was also lower than that in the SH group (2127.5 ± 153.6 mmol/l·min vs. 2445.0 ± 148.1 mmol/l·min, *P* < 0.001).

### Effects on FXR target mRNA expression

**Figure 6:**
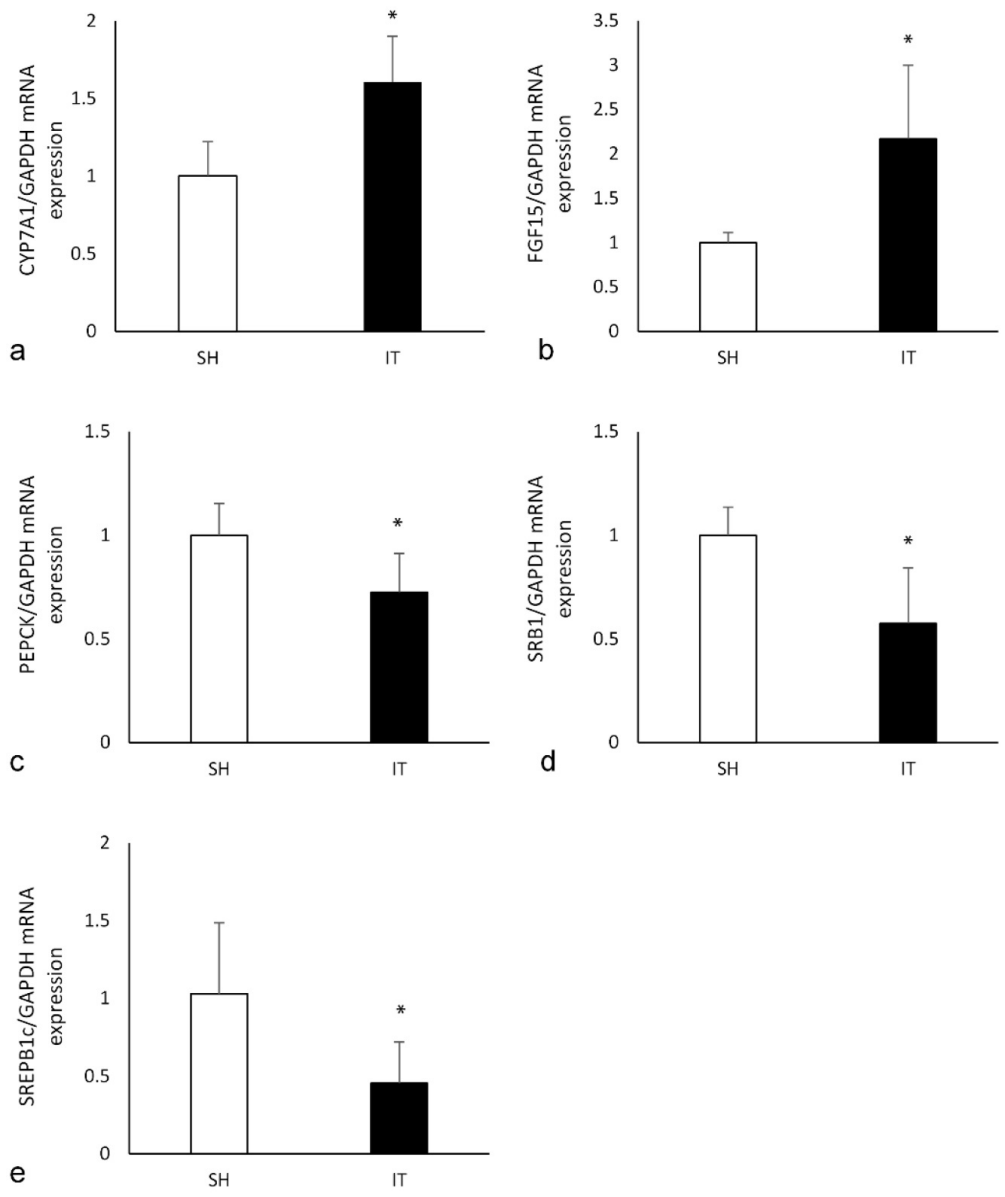
the expression of key FXR target genes. a, the expression of CYP7A1 mRNA in liver. The FXR activation could reduce the expression of CYP7A1. b, the expression of FGF15 mRNA in ileum. The FGF15 expression is inhibited by activated FXR. c, the expression of PEPCK mRNA in liver. Phosphoenolpyruvate carboxykinase is a key enzyme in the gluconeogenesis pathway d, the expression of SRB1 mRNA in ileum. Scavenger receptor class B type 1 is involved in cholesterol transportation. e, the expression of SREPB1c mRNA in liver. Sterol regulatory element binding proteins induction causes lipogenesis, hypertriglyceridemia and steatohepatitis. FXR, farnesoid X receptor. IT, ileum transposition. SH, sham ileum transposition.

In the liver tissue of the IT group, the expression of CYP7A1 mRNA was significantly higher than that in the SH group (*P* < 0.001, Figure 6), and the expression of PEPCK mRNA (*P* = 0.004) and SREPB1c mRNA (*P* = 0.005) were significantly lower than that in the SH group. In the ileal tissue of the IT group, the expression of FGF15 mRNA was significantly higher than that in the SH group (*P* = 0.001), and the expression of SRB1 mRNA was lower than that in the SH group (*P* = 0.001).

## Discussion

IT procedures, as a bariatric surgery, could effectively improve glucose metabolism, which is in agreement with previous findings 10, 25. The level of FPG and AUC_OGTT_ in the IT group decreased significantly after surgery and was lower than those in the SH group at postoperative week 20. We previously proposed a role of BAs in the improvement of glucose metabolism but did not analyze BA profiles changes or investigate the downstream mechanism 10. In this study, 17 kinds of BAs were measured and compared, and the level of β-MCA, T-α-MCA and T-β-MCA in plasma in the IT group was higher than those in the SH group. Moreover, we found that the level of TCA and TDCA in plasma was lower than those in the SH group. The BA profiles in liver and ileum tissues were also analyzed, the level of GUDCA, T-α-MCA and T-β-MCA in liver and ileum tissues in the IT group was higher than that in the SH group. BA profiles changes indicate possible roles in the improvement of glucose metabolism after IT.

BAs could decompose lipid food and involve the absorption of lipid. More importantly, they could act as signaling molecules connecting neighboring regions and distant tissues by blood circulation. The gut-liver axis, gut-brain axis, gut-liver-brain axis, and parts of the immune system has received considerable attention. Many experiments have investigated in the direct and indirect pathways among organs and tissues, including the vagus nerve and BA signaling pathways 26. Various peptides, hormones and bile acids integrate gut, brain and peripheral tissues, and regulate the energy homeostasis and glucose metabolism 27. These communications coordinate the regulation of nutrients intake, glycogen synthesis and lysis, energy expenditure. It is worth noting that secondary bile acids were strongly associated with neuropathological behaviors, especially the DCA and its glycine- and taurine-conjugated forms 26. By contrast, UDCA has been demonstrated to play neuroprotective roles by suppressing inflammation and apoptosis in rat models 28. In our results, the low level of TDCA in plasma and high level of GUDCA might involve in the improvement of glucose metabolism. BAs activates their receptors, like a nuclear receptor FXR. A high level of FXR is observed in the ileum and liver, and the pancreas, kidney, cardiovascular system and brain tissue also express FXR 7, 8. CA, CDCA, LCA and DCA are natural agonists of FXR 11. TDCA and TCA are thought to also have little FXR activity 29. UDCA, T-α-MCA, T-β-MCA and TUDCA are serum FXR antagonist 30. And T-β-MCA has fair intestinal stability because of its' resistance to bacterial bile salt hydrolase 31.

FXR activation suppresses the expression of cholesterol 7α-hydroxylase that is a rate-limiting enzyme in BA biosynthesis. And overexpression of cholesterol 7α-hydroxylase could cause weight reduction, improvement of glucose tolerance and insulin resistance, protection from dyslipidemia and inflammation 32. In addition, BA-FXR signaling also represses phosphoenolpyruvate carboxykinase and glucose 6-phosphatase to decrease liver gluconeogenesis accompanied by the induction of hepatic glycogen synthesis 33. The glucose metabolic derangements and obesity would be remarkable alleviated. In our study, the expression of CYP7A1 mRNA in the liver of the IT group was higher than that in the SH group, and the expression of PEPCK mRNA was significantly lower than that in the SH group. Phosphoenolpyruvate carboxykinase (PEPCK) is a key enzyme in the gluconeogenesis pathway, the expression level of PEPCK mRNA regulates the oxidation and synthesis of glucose. Therefore, the glucose metabolism in the IT group is improved. Scavenger receptor class B type 1 (SRB1) is involved in cholesterol transportation and Sterol regulatory element binding proteins (SREBP-1c) induction causes lipogenesis, hypertriglyceridemia and steatohepatitis 34. Lower expression of SRB1 mRNA and SREBP-1c mRNA in the IT group could explain the reason of slow body weight gain.

That FXR inhibition could improve glucose metabolism was also verified by oral inhibitor test at postoperative week 18. The inhibition efficiency of FXR was verified by previous report 18. After oral FXR inhibition, glucose metabolism in the SH group rats was improved. However, there was no significant difference in the IT group. In previous investigations, oral administration of FXR antagonist, T-β-MCA derivative or glycine-β-MCA, was found to decrease murine FXR signals in the ileum, that resulted in improvement of insulin resistance, obesity and liver steatosis associated with lower level of serum ceramide levels 18. Mice with intestinal-selective FXR inhibition showed protection from the development of glucose intolerance and obesity 18. Deletion of FXR in liver of mice improved glucose tolerance and fatty acid metabolism and reversed the aging phenotype of increased adiposity and impaired glucose sensing 35. Deletion of FXR in obese mice underwent bariatric surgery exhibited impaired weight loss and deterioration of glucose tolerance improvement 36.

When treated with an FXR agonist, insulin resistance and lower GLP-1 levels were observed 14. Although a report showed that the FXR agonist fexaramine improved insulin and glucose tolerance 37, the results demonstrated that modulating the gut microbiota increased level of taurolithocholic acid. High level of taurolithocholic acid enhanced the secretion of fibroblast growth factors 15 and 21, which could improve insulin resistance. Because powerful and extensive activation of FXR has many side effects 11, different agonists might affect different tissue FXRs and aspects, which leads to complex alterations in glucose metabolism and energy homeostasis. Collectively, most studies demonstrate that intestinal FXR activation has a detrimental effect on glucose metabolism. Our results approved the deterioration of glucose metabolism of FXR activation, and the expression of FGF15 mRNA in the ileal tissue of IT group was significantly higher than that in the SH group.

There are a few limitations in our study. One major limitation relates to the fact that rats and humans are inherently different in many aspects, such as BA biology and compositions. Even in the same animal species, the bile acid composition is different between individuals, as transformation and reabsorption of bile acid varies before or during diet administration 38. Besides, the limited model number of each group and lack of control without surgery may hinder accurate interpretation of results. Large scale randomized controlled study about causal relationship and the therapeutic efficiency of bile acids is still needed in future. At least, our results revealed significant changes of bile acid profiles after IT and possible role of bile acid on glucose metabolism. Insight into the roles of bile acids and its receptors and explore less invasive medicine might provide a new treatment strategy for T2DM.

## Conclusions

Our results show that bile acid profiles change significantly after IT, FXR activation might has a detrimental effect on glucose metabolism.

## References

[1] Safiri S, Karamzad N, Kaufman JS, Bell AW, Nejadghaderi SA, Sullman MJM (2022). Prevalence, Deaths and Disability-Adjusted-Life-Years (DALYs) Due to Type 2 Diabetes and Its Attributable Risk Factors in 204 Countries and Territories, 1990-2019: Results From the Global Burden of Disease Study 2019. Front Endocrinol (Lausanne).

[2] Yan K, Chen W, Zhu H, Lin G, Pan H, Li N (2018). Ileal Transposition Surgery Decreases Fat Mass and Improves Glucose Metabolism in Diabetic GK Rats: Possible Involvement of FGF21. Front Physiol.

[3] Ahn CH, Choi EH, Oh TJ, Cho YM (2020). Ileal Transposition Increases Pancreatic beta Cell Mass and Decreases beta Cell Senescence in Diet-Induced Obese Rats. Obes Surg.

[4] Cohen R, Caravatto PP, Petry TZ (2016). Innovative metabolic operations. Surg Obes Relat Dis.

[5] Kohli R, Kirby M, Setchell KD, Jha P, Klustaitis K, Woollett LA (2010). Intestinal adaptation after ileal interposition surgery increases bile acid recycling and protects against obesity-related comorbidities. Am J Physiol Gastrointest Liver Physiol.

[6] Xue R, Su L, Lai S, Wang Y, Zhao D, Fan J (2021). Bile Acid Receptors and the Gut-Liver Axis in Nonalcoholic Fatty Liver Disease. Cells.

[7] Hou Y, Fan W, Yang W, Samdani AQ, Jackson AO, Qu S (2019). Farnesoid X receptor: An important factor in blood glucose regulation. Clin Chim Acta.

[8] Yang F, Tang X, Ding L, Zhou Y, Yang Q, Gong J (2016). Curcumin protects ANIT-induced cholestasis through signaling pathway of FXR-regulated bile acid and inflammation. Sci Rep.

[9] Goncalves D, Barataud A, De Vadder F, Vinera J, Zitoun C, Duchampt A (2015). Bile Routing Modification Reproduces Key Features of Gastric Bypass in Rat. Ann Surg.

[10] Chen W, Yin H, Zhang N, Liu W, Qu Q, Xiao J (2021). Improvement of Postprandial Lipid Metabolism After Ileal Transposition in Non-obese Diabetic Rats. Obes Surg.

[11] Zhang C, Wang Z, Feng Q, Chen WD, Wang YD (2020). Farnesoid X receptor: a potential therapeutic target in multiple organs. Histol Histopathol.

[12] Cipriani S, Mencarelli A, Palladino G, Fiorucci S (2010). FXR activation reverses insulin resistance and lipid abnormalities and protects against liver steatosis in Zucker (fa/fa) obese rats. J Lipid Res.

[13] Fang S, Suh JM, Reilly SM, Yu E, Osborn O, Lackey D (2015). Intestinal FXR agonism promotes adipose tissue browning and reduces obesity and insulin resistance. Nat Med.

[14] Watanabe M, Horai Y, Houten SM, Morimoto K, Sugizaki T, Arita E (2011). Lowering bile acid pool size with a synthetic farnesoid X receptor (FXR) agonist induces obesity and diabetes through reduced energy expenditure. J Biol Chem.

[15] Galan BSM, Serdan TDA, Rodrigues LE, Manoel R, Gorjao R, Masi LN (2022). Reviewing physical exercise in non-obese diabetic Goto-Kakizaki rats. Braz J Med Biol Res.

[16] Burgos-Ramos E, Canelles S, Frago LM, Chowen JA, Arilla-Ferreiro E, Argente J (2016). Improvement in glycemia after glucose or insulin overload in leptin-infused rats is associated with insulin-related activation of hepatic glucose metabolism. Nutr Metab (Lond).

[17] Yang T, Shu T, Liu G, Mei H, Zhu X, Huang X (2017). Quantitative profiling of 19 bile acids in rat plasma, liver, bile and different intestinal section contents to investigate bile acid homeostasis and the application of temporal variation of endogenous bile acids. J Steroid Biochem Mol Biol.

[18] Jiang C, Xie C, Lv Y, Li J, Krausz KW, Shi J (2015). Intestine-selective farnesoid X receptor inhibition improves obesity-related metabolic dysfunction. Nat Commun.

[19] Akwabi-Ameyaw A, Bass JY, Caldwell RD, Caravella JA, Chen L, Creech KL (2008). Conformationally constrained farnesoid X receptor (FXR) agonists: Naphthoic acid-based analogs of GW 4064. Bioorg Med Chem Lett.

[20] Pan J, Ouyang X, Jin Q, Wang W, Xie J, Yu B (2022). Hypolipidemic effect of ethanol extract from Chimonanthus nitens Oliv. leaves in hyperlipidemia rats via activation of the leptin/JAK2/STAT3 pathway. Mol Med.

[21] Luo M, Yan J, Wu L, Wu J, Chen Z, Jiang J (2021). Probiotics Alleviated Nonalcoholic Fatty Liver Disease in High-Fat Diet-Fed Rats via Gut Microbiota/FXR/FGF15 Signaling Pathway. J Immunol Res.

[22] Yan Y, Sha Y, Huang X, Yuan W, Wu F, Hong J (2019). Roux-en-Y Gastric Bypass Improves Metabolic Conditions in Association with Increased Serum Bile Acids Level and Hepatic Farnesoid X Receptor Expression in a T2DM Rat Model. Obes Surg.

[23] Barlow NJ, Phillips SL, Wallace DG, Sar M, Gaido KW, Foster PM (2003). Quantitative changes in gene expression in fetal rat testes following exposure to di(n-butyl) phthalate. Toxicol Sci.

[24] Kojima M, Nemoto K, Murai U, Yoshimura N, Ayabe Y, Degawa M (2002). Altered gene expression of hepatic lanosterol 14alpha-demethylase (CYP51) in lead nitrate-treated rats. Arch Toxicol.

[25] Cummings BP, Strader AD, Stanhope KL, Graham JL, Lee J, Raybould HE (2010). Ileal interposition surgery improves glucose and lipid metabolism and delays diabetes onset in the UCD-T2DM rat. Gastroenterology.

[26] Tung TH, Chen YC, Lin YT, Huang SY (2022). N-3 PUFA Ameliorates the Gut Microbiota, Bile Acid Profiles, and Neuropsychiatric Behaviours in a Rat Model of Geriatric Depression. Biomedicines.

[27] Wachsmuth HR, Weninger SN, Duca FA (2022). Role of the gut-brain axis in energy and glucose metabolism. Exp Mol Med.

[28] Abdelkader NF, Safar MM, Salem HA (2016). Ursodeoxycholic Acid Ameliorates Apoptotic Cascade in the Rotenone Model of Parkinson's Disease: Modulation of Mitochondrial Perturbations. Mol Neurobiol.

[29] Dossa AY, Escobar O, Golden J, Frey MR, Ford HR, Gayer CP (2016). Bile acids regulate intestinal cell proliferation by modulating EGFR and FXR signaling. Am J Physiol Gastrointest Liver Physiol.

[30] Lim T, Lee K, Kim RH, Cha KH, Koo SY, Moon EC (2022). Black raspberry extract can lower serum LDL cholesterol via modulation of gut microbial composition and serum bile acid profile in rats fed trimethylamine-N-oxide with a high-fat diet. Food Sci Biotechnol.

[31] Gonzalez FJ, Jiang C, Xie C, Patterson AD (2017). Intestinal Farnesoid X Receptor Signaling Modulates Metabolic Disease. Dig Dis.

[32] Shapiro H, Kolodziejczyk AA, Halstuch D, Elinav E (2018). Bile acids in glucose metabolism in health and disease. J Exp Med.

[33] Zhang Y, Lee FY, Barrera G, Lee H, Vales C, Gonzalez FJ (2006). Activation of the nuclear receptor FXR improves hyperglycemia and hyperlipidemia in diabetic mice. Proc Natl Acad Sci U S A.

[34] Lewandowski CT, Khan MW, BenAissa M, Dubrovskyi O, Ackerman-Berrier M, LaDu MJ (2021). Metabolomic analysis of a selective ABCA1 inducer in obesogenic challenge provides a rationale for therapeutic development. EBioMedicine.

[35] Kim KH, Choi S, Zhou Y, Kim EY, Lee JM, Saha PK (2017). Hepatic FXR/SHP axis modulates systemic glucose and fatty acid homeostasis in aged mice. Hepatology.

[36] Ryan KK, Tremaroli V, Clemmensen C, Kovatcheva-Datchary P, Myronovych A, Karns R (2014). FXR is a molecular target for the effects of vertical sleeve gastrectomy. Nature.

[37] Pathak P, Xie C, Nichols RG, Ferrell JM, Boehme S, Krausz KW (2018). Intestine farnesoid X receptor agonist and the gut microbiota activate G-protein bile acid receptor-1 signaling to improve metabolism. Hepatology.

[38] Thakare R, Alamoudi JA, Gautam N, Rodrigues AD, Alnouti Y (2018). Species differences in bile acids I. Plasma and urine bile acid composition. J Appl Toxicol.

